# Uveitis and blindness in a closed herd of Equidae following leptospiral infection

**DOI:** 10.3389/fvets.2024.1504990

**Published:** 2025-01-06

**Authors:** J. Gerras, K. Young, D. Roberts, G. Waldman, J. H. Salmon, B. C. Gilger

**Affiliations:** ^1^Department of Clinical Sciences, North Carolina State University, Raleigh, NC, United States; ^2^Rivendell Mobile Large Animal, Advance, NC, United States

**Keywords:** horse, uveitis, leptospirosis, environment, susceptibility

## Abstract

**Objective:**

To describe the ocular findings, chronology of disease, and serum leptospiral titers in a group of horses, mules, and donkeys following an outbreak of leptospirosis.

**Methods:**

Fifty Equidae in central North Carolina had ophthalmic examinations and serum leptospiral microscopic agglutination test (MAT) titers performed every 3–6 months for 24 months followed by a final examination at 34 months.

**Results:**

Throughout the nearly three-year study period, 17 horses (34%; 17/49 horses) developed signs of uveitis; 20 eyes (20/34; 58.8%) of these 17 horses were visual at the initial examination, but only four eyes (11.8%) remained visual at the final examination. Serum titers (serogroups Pomona and Bratislava) in horses with uveitis were significantly elevated compared to Equidae without uveitis (*p* < 0.02). In the 32 horses, donkeys, and mules that did not develop uveitis, a subgroup of 11 horses and one donkey had negative or low serum leptospiral titers (titers ≤1:800) while a second subgroup of 16 horses, three mules, and one donkey had high leptospiral titers (>1:800) but never developed uveitis. Water sources in the pasture were found to have high levels of leptospira.

**Conclusion:**

Approximately 1/3 of horses on a farm exposed to Leptospira developed uveitis and blindness. Serum titers to *L.* Pomona and *L.* Bratislava were significantly elevated in horses with uveitis. However, despite exposure, some horses, even with very high serum titers, never developed ocular disease. These data indicates that further research is warranted to investigate the genetic and immunological aspects of the pathogenesis and susceptibility of leptospiral-associated uveitis.

## 1 Introduction

The association between leptospiral infection and equine uveitis in geographical areas of the United States is well known. In a recent study, 46 % of horses with uveitis in North Carolina had positive serum or aqueous humor titers to *Leptospira (L)* serogroups Pomona or Grippotyphosa (1–8). In other studies, most horses with uveitis in Upstate New York and Minnesota were seropositive for *L.* Pomona (6, 8). Furthermore, polymerase chain reaction (PCR) testing of aqueous or vitreous humor has been positive for leptospiral DNA in up to 70% of horses with uveitis in the Southeastern US and California (1, 2, 9). The immunopathogenesis of recurrent uveitis in horses has been thoroughly studied, and the phenomenon of molecular mimicry occurs where there is an immunological association between leptospiral antigens and equine ocular antigens, resulting in recognition and immunologic response to retinal self-antigens (7, 10–14). This immunopathogenesis helps to explain the development of uveitis and the subsequent episodes of recurrent uveitis after leptospiral infection (11, 12, 14). Further, biofilm formation may also play a role in the development of uveitis in horses following leptospiral infection (15).

Animals are exposed to leptospira when contaminated water or food containing urine from an infected host-adapted species enter the body via the mucous membranes or skin lesions. In horses, leptospiral exposure is common following heavy rains or flooding in which horses are exposed to stagnant water (3, 16, 17). Leptospiral infections in horses have been associated with abortion, acute renal failure, and neonatal disease, but most commonly, horses develop transient fever, malaise, and jaundice, which are rarely observed or reported. Thus, signs of acute disease are primarily subclinical. However, uveitis has been reported to develop in horses up to 24 months after acute infection with leptospira (5, 18). In one report of natural exposure and disease in which *L.* Pomona was isolated, horses developed uveitis 18–24 months after signs of acute leptospirosis (5). In another study, a group of ponies with experimentally-induced *L. interrogans* serogroup Pomona infection developed ocular inflammation in 61% of eyes within 15 months of the initial leptospiral exposure (18).

These previous studies support the association between equine uveitis and Leptospirosis, especially with *L.* Pomona and Grippotyphosa infections (1, 2, 18). However, the natural course of the disease and development of uveitis in horses following leptospiral infection were reported over 40 to 60 years ago, and these reports had limited follow-up, no serial leptospiral serum titer information, and only brief descriptions of the ophthalmic examination findings (5, 18, 19). Thus, the prevalence of ocular disease, the time course, and the outcome following spontaneous leptospiral infections are unknown. Furthermore, susceptibility factors and time course related to leptospiral infection, development of uveitis, and progression to blindness in horses are also unknown. Identifying sources of infection and determining how to prevent acute and chronic disease are important factors that need additional study.

The purpose of this prospective descriptive cohort study was to report the ocular findings, the chronology of disease, and serum leptospiral titer changes over time in a diverse group of horses, mules, and donkeys on a single farm following an outbreak of leptospirosis with an emphasis on investigating risk factors and susceptibility of these animals to developing ocular disease and blindness.

## 2 Methods

### 2.1 Animals studied

A prospective longitudinal cohort observational study (20–22) followed a group of horses on a single farm from October 2021 through August 2024 after an outbreak of leptospirosis in early 2021. The equine ophthalmology service at NC State University was consulted in October 2021 regarding the development of uveitis and blindness in multiple horses in a herd located in central North Carolina (Mocksville, NC). The farm is a rescue facility with 49 Equidae (horses, donkeys, and mules) of diverse breeds and ages (Supplementary Table S1). The animals were kept in a large single pasture, fed primarily grass, and supplemented with hay. All horses, mules, and donkeys had access to the same water sources throughout the pasture. The animals received annual core vaccinations (equine influenza, equine herpesvirus-1 and −4, tetanus, rabies, Eastern and Western equine encephalomyelitis, and West Nile) (23) and anthelmintics (ivermectin) but did not receive routine therapeutics. A few horses were used for pleasure riding, but most were not handled. The farm owners observed that several horses lost appetite in January, February, and March 2021 and were lethargic, which lasted for several days but then spontaneously resolved.

The NC State Ophthalmology service initially examined the horses on the farm in early October 2021 (i.e., 7 to 9 months following initial signs of illness). Ocular examinations, consisting of slit lamp biomicroscopy (Kowa SL-17, Kowa USA, Torrence, CA) and indirect ophthalmoscopy (Keeler Vantage, Keeler USA, Malvern, PA) with lens (2.2 Panretinal lens, Volk, Mentor, OH) were performed by a board-certified veterinary ophthalmologist on all horses in October 2021. Examinations were repeated approximately every 3–6 months through October 2023, with a final follow-up examination performed in August 2024, nearly 3 years after the initial assessment. Other ocular diagnostic methods were not performed due to the logistics of examining and collecting samples from a large number of horses on a farm. The owners of the horses provided written consent to examine and collect samples from the horses. If horses showed signs of discomfort, they were treated symptomatically by the local veterinarian with systemic nonsteroidal anti-inflammatory medications (NSAIDs), such as flunixin meglumine or phenylbutazone. Topical ocular application of corticosteroids, NSAIDs, or atropine was not done. This study was observational only, and treatment decisions were made by the referring veterinarian and owner. Enrollment into the study did not dictate therapy. This study was approved and monitored by the North Carolina State University institutional animal care and use committee (IACUC, # 24–299).

### 2.2 Inclusion criteria and clinical descriptions

Horses were considered to have active uveitis if they had clinical signs of aqueous flare, miosis, with or without anterior chamber fibrin or cells, iris hyperemia, and vitreous cells (1, 24–27). They were considered to have chronic uveitis if one or several of the following was observed: corpora nigra atrophy, posterior synechia, corneal edema, iris hyperpigmentation, iris fibrosis, cataract, vitreous degeneration, or retinal degeneration (1, 24–27). Horses were considered blind if they had no menace response, dazzle reflex, and negative consensual pupillary light reflex. Phthisis bulbi was diagnosed when the eye had chronic signs of uveitis, blindness, plus reduced anterior chamber and globe size. Horses that developed uveitis were classified at each examination time as having active uveitis (Active), chronic uveitis with vision (Quiescent / chronic uveitis [Visual]), chronic uveitis with blindness (Quiescent / chronic uveitis [Blind]), or having phthisis bulbi (Phthisis).

### 2.3 Laboratory testing

Serial leptospiral microscopic agglutination tests (MAT) were performed on serum collected from each horse every 3–6 months for 2 years at the time of the ophthalmic examinations, then a final time point 34 months after the initial examination for the most prevalent serovars in North America as determined by the National Veterinary Services Laboratory (NVSL; Ames, IA) and included *L.* Pomona, Icterohaemorrhagiae, Canicola, Grippotyphosa, Hardjo, Bratislava, and Autumnalis (North Carolina Veterinary Diagnostic Laboratory System; Rollins Laboratory, Raleigh, NC). All titers were obtained in a single MAT reaction (including co-agglutination titers). Leptospiral MAT titer levels of ≤1:800 were interpreted as low titers and suggestive of a previous exposure. In contrast, serum MAT levels of ≥1:1600 were considered very high titers and consistent with a recent infection.^1^ More definitive diagnoses of leptospiral-induced uveitis through MAT testing or PCR of ocular fluids were not performed because of the logistics of collecting the samples in the field.

In December 2022 and August 2024, one-liter water samples were collected from each water source to which the animals had access on the farm. Samples were collected from two separate wells, a creek, a centrally located pond, and the county public water supply. Using qPCR, a commercial environmental laboratory (EMSL Analytical, Inc., Cinnaminson, NJ) tested the water samples for leptospiral content.

### 2.4 Statistical analysis

Linear mixed models (LMM) were constructed to assess differences in leptospiral titer values between animals with and without uveitis across time during the 34 months of evaluation. For leptospiral titer values, the titer dilution values were used as the longitudinal outcome for the mixed-effect model. The fixed effects include uveitis status and time points (month). Horse was included as a random effect to account for multiple measurements from the same animal. Descriptive analyses were conducted to summarize as mean ± standard deviation (SD), whereas categorical data were summarized as counts (n) and percentages (%). Comparisons of differences in genders and breeds between horses with and without uveitis were analyzed using Pearson’s Chi-Square test. Statistical significance was set at *p* < 0.05. Statistical analyses were performed using a commercial software package (JMP Pro v. 16.0.0, Cary, North Carolina). Figures were created using a commercially available software package (GraphPad Prism 10.2.3).

## 3 Results

### 3.1 Animals studied

In total, 44 horses, three mules, and two donkeys (total of *n* = 49 Equidae) were examined by the NCSU ophthalmology service in October 2021, 7 to 9 months after horses on the farm exhibited signs of illness, consisting predominantly of decreased activity and appetite. The ophthalmic examinations were conducted approximately 2 months after the owners first observed signs of blindness in several horses. The horses on the farm represented a variety of breeds, including thirteen quarter horses, nine mixed breed ponies, seven draft or draft crosses, five miniature horses, two Tennessee Walking Horses, three Thoroughbreds, two Norwegian fjords, and one each of a Haflinger, Appaloosa, and Mustang. See Supplementary Table S1 for a complete list of breeds, sexes, and ages.

Throughout the nearly three-year study period, 17 horses (34% of 49 total Equidae) developed signs of uveitis in one or both eyes. In contrast, the remainder of the horses, mules, or donkeys (*n* = 32) did not have evidence of uveitis on examination at any time. During the study period, three horses died in the uveitis group, all following the May 2022 examination, and five horses died in the non-uveitis group, two after the September 2022 examination and three after the May 2023 examination. Therefore, their data is not included after these time points. Their deaths were considered age-related and not associated with systemic infectious or ocular disease. One horse had left the farm briefly for another stable and was not available for examination during the first two periods (October 2021 and February 2022), but its data was included starting at the May 22 examination (Month 7) for the remainder of the study (Supplementary Tables S1, S2). All of the animals that developed uveitis were horses, including ten mares and seven geldings, and their mean age at the time of the initial visit was 19.4 +/−SD 4.2 years. The remainder of the horses (*n* = 21), miniature horses (*n* = 5), donkeys (*n* = 2), and 3 mules (*n* = 4) on the farm did not exhibit signs of acute or chronic uveitis throughout the 3-year follow-up period. The 32 animals that did not develop uveitis had a mean age of 17.5 +/−SD 6.4 years at the start of the study and consisted of 14 mares and 18 geldings. There was no significant difference between age (*p* = 0.2755) and sex (*p* = 0.2415) in animals that did or did not develop uveitis. Horse breeds were not significantly different in the uveitis and non-uveitis groups (see Supplementary Table S1); however, miniature horses (*n* = 5), mules (*n* = 4), and donkeys (*n* = 2) did not develop uveitis at any point during the study and were significantly less likely to get uveitis than horses (*p* = 0.0056).

**Figure 1:**
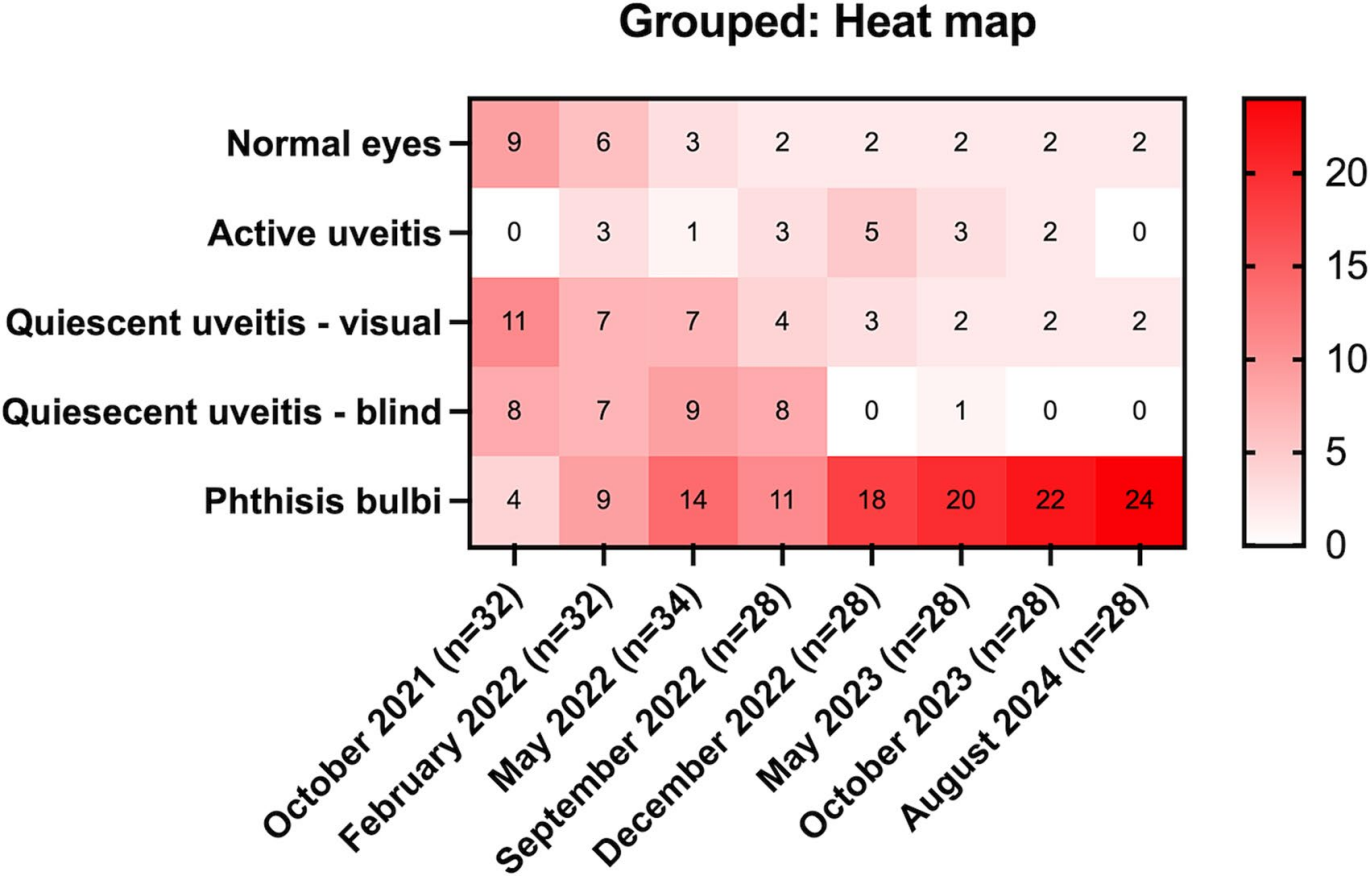
Heat map of eyes with uveitis (*n* = 17 horses). Over 3 years, in equidae that developed uveitis, the number of normal eyes decreased from 9 (28.1%) to 2 (7.1%), the number of eyes with quiescent uveitis (but remained visual) decreased from 11 (34.4%) to 1 (3.6%), and the number of eyes with phthisis bulbi increased from 4 (12.5%) to 24 (82.1%).

**Figure 2:**
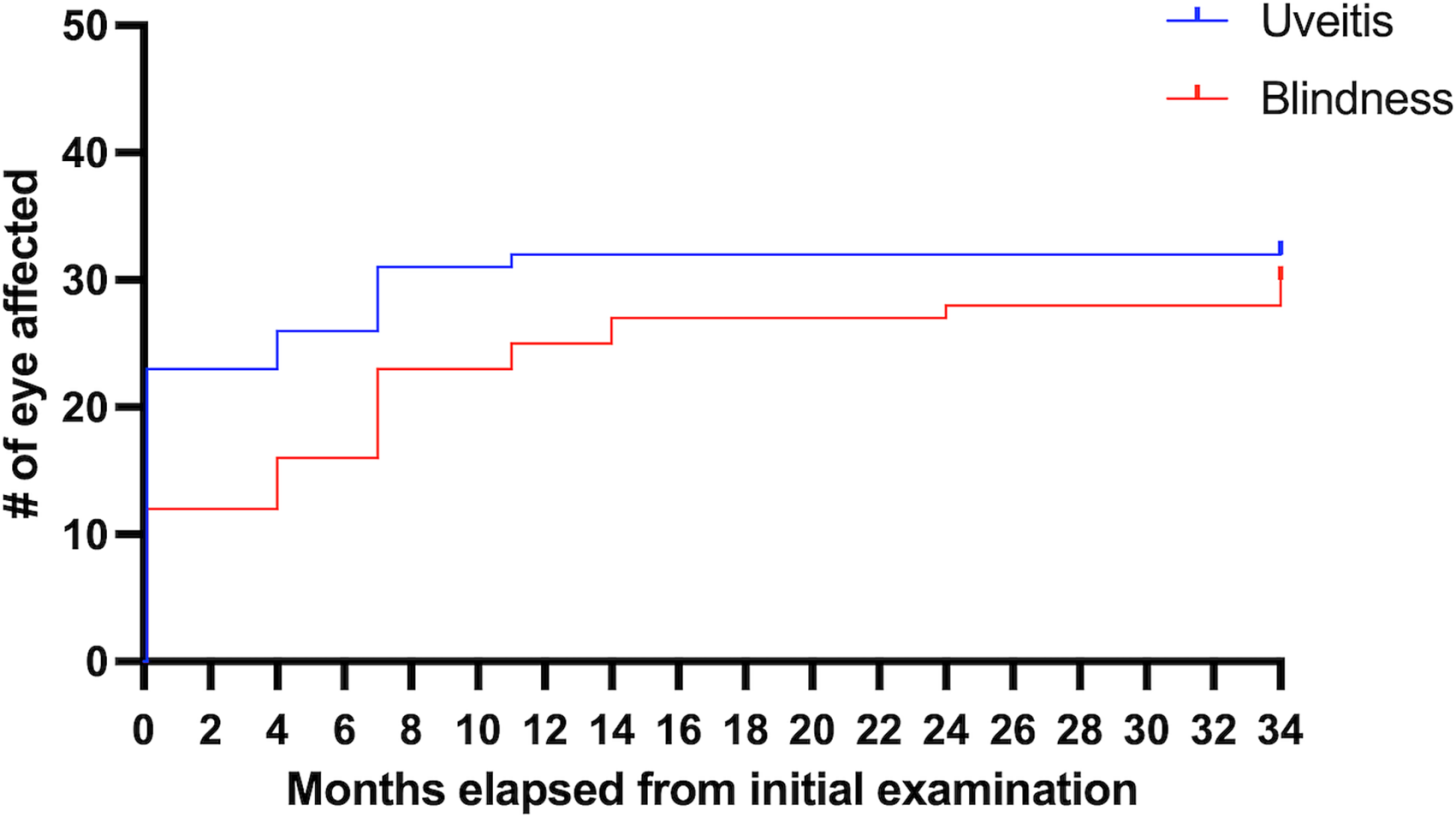
Percentages of eyes of horses on the farm with uveitis and blindness over the 34 months follow-up. Of all the horses on the farm (those with and without uveitis), this represents 81.6% of the eyes (80 of 98 eyes) were initially visual but only 70.4% (69 of 98 eyes) remained visual after 34 months of observation.

Of the 17 horses that developed uveitis, 16 horses (32 eyes) were initially examined in October 2021 (Month 0), and of these, nine horses developed bilateral disease, and seven were unilateral. Eleven eyes (32.4%; 11/32 eyes) had signs of chronic uveitis but were visual (Quiescent/chronic uveitis [Visual]), eight eyes (25%; 8/32 eyes) had chronic uveitis but were blind (Quiescent / chronic uveitis [Blind]), four eyes had phthisis bulbi (12.5%; 4/32 eyes), no eyes had active uveitis, and nine eyes (28.1%; 9/32 eyes) were considered normal (Table 1, Figure 1, Supplementary Table S2). Over the subsequent three years, the number of normal eyes in this group of horses (with uveitis) decreased from 9 (28.1%; 9/32 eyes) to 2 (7.1%; 2/28 eyes), the number of eyes with quiescent uveitis (but remained visual) decreased from 11 (34.4%; 11/32 eyes) to 2 (7.1%; 2/28 eyes) and the number of eyes with phthisis bulbi increased from 4 (12.5%; 4/32 eyes) to 24 (85.7%; 24/28 eyes) (Table 1, Figure 1, Supplementary Table S2). Overall, in the horses (*n* = 17) that developed uveitis throughout the study, 20 of their eyes (62.5%; 20/32 eyes) were considered visual at Month 0, but only four of their eyes (14.3%, 4/28 eyes) remained visual at Month 34 (Table 1, Figure 1). In all Equidae (uveitis and non-uveitis), at the beginning of the study period, 89.8% of the eyes (88 of 98 eyes) were initially visual, but only 72.45% (71 of 98) remained visual after 34 months of observation (Figure 2). These data demonstrate the poor visual prognosis in horses with presumed leptospiral-associated uveitis. The initial examination (Month 0) in this study was conducted 7 to 9 months after the clinical signs of primary infection, and the animals were followed for 34 months. The last case of new uveitis (normal eye developing uveitis) was observed in September 2022 (Month 11), and this eye had active uveitis for over a year and progressed to phthisis by August 2024 (Month 34) (Figure 2; Supplementary Table S2). These findings suggest that uveitis can develop up to 17–19 months after the primary leptospiral infection and vision loss can be progressive for up to 34 months or longer in horses.

**Table 1:** Status of eyes in horses that developed uveitis per examination time.

	Normal eyes	Active uveitis	Quiescent / chronic uveitis (Visual)	Quiescent / chronic uveitis (Blind)	Phthisis bulbi
Dates	#eyes (% of total)				
October 2021 (Month 0) (*n* = 16 horses / 32 eyes)	9 (28.1%)	0 (0%)	11 (34.4%)	8 (25%)	4 (2.5%)
February 2022 (Month 4) (*n* = 16 horses / 32 eyes)	6 (18.8%)	3 (9.4%)	7 (21.9%)	7 (21.9%)	9 (28.1%)
May 2022 (Month 7) (*n* = 17 horses, 34 eyes)	3 (8.8%)	1 (2.9%)	7 (20.6%)	9 (26.5%)	14 (41.2%)
September 2022 (Month 11) (*n* = 14 horses, 28 eyes)	2 (7.1%)	3 (10.7%)	4 (14.3%)	8 (28.6%)	11 (39.3%)
December 2022 (Month 14) (*n* = 14 horses, 28 eyes)	2 (7.1%)	5 (17.9%)	3 (10.7%)	0 (0%)	18 (64.3%)
May 2023 (Month 19) (*n* = 14 horses, 28 eyes)	2 (7.1%)	3 (10.7%)	2 (7.1%)	1 (3.6%)	20 (71.4%)
October 2023 (Month 24) (*n* = 14 horses, 28 eyes)	2 (7.1%)	2 (7.1%)	1 (3.6%)	0 (0%)	23 (82.1%)
August 2024 (Month 34) (*n* = 14 horses, 28 eyes)	2 (7.1%)	0 (0%)	2 (7.1%)	0 (0%)	24 (85.7%)

### 3.2 Active uveitis and recurrence of inflammation

Over the nearly 3 years of observation, nine eyes (26%; 9/34 total eyes in 17 uveitis horses) had signs of active inflammation: 3 were new cases of uveitis in previously normal eyes, and 6 were recurrences of uveitis (in eyes with previous chronic signs of uveitis). One eye had a second active episode recurrence 10 months after the first active episode (Table 1, Supplementary Table S2). Six of the nine active episodes lasted 3 months or less, one was active for 6 months or less, and one case of bilateral uveitis was active for more than 1 year. Active uveitis was observed in 3 eyes at the 4-month exam, in 1 eye at the 7-month exam, in 3 eyes at the 11-month exam, in 5 eyes at the 14-month exam, in 3 eyes at the 19-month exam, and in 2 eyes at the 24-month exam (Table 1, Figure 2, Supplementary Table S2). As mentioned, the last case of new active uveitis (normal eye developing uveitis) was observed in September 2022, at the 11-month examination. Active inflammation was not observed in any eye after the 24-month examination nor following the development of phthisis bulbi (Table 1, Figure 1, Supplementary Table S2).

### 3.3 Leptospiral titers

**Figure 3:**
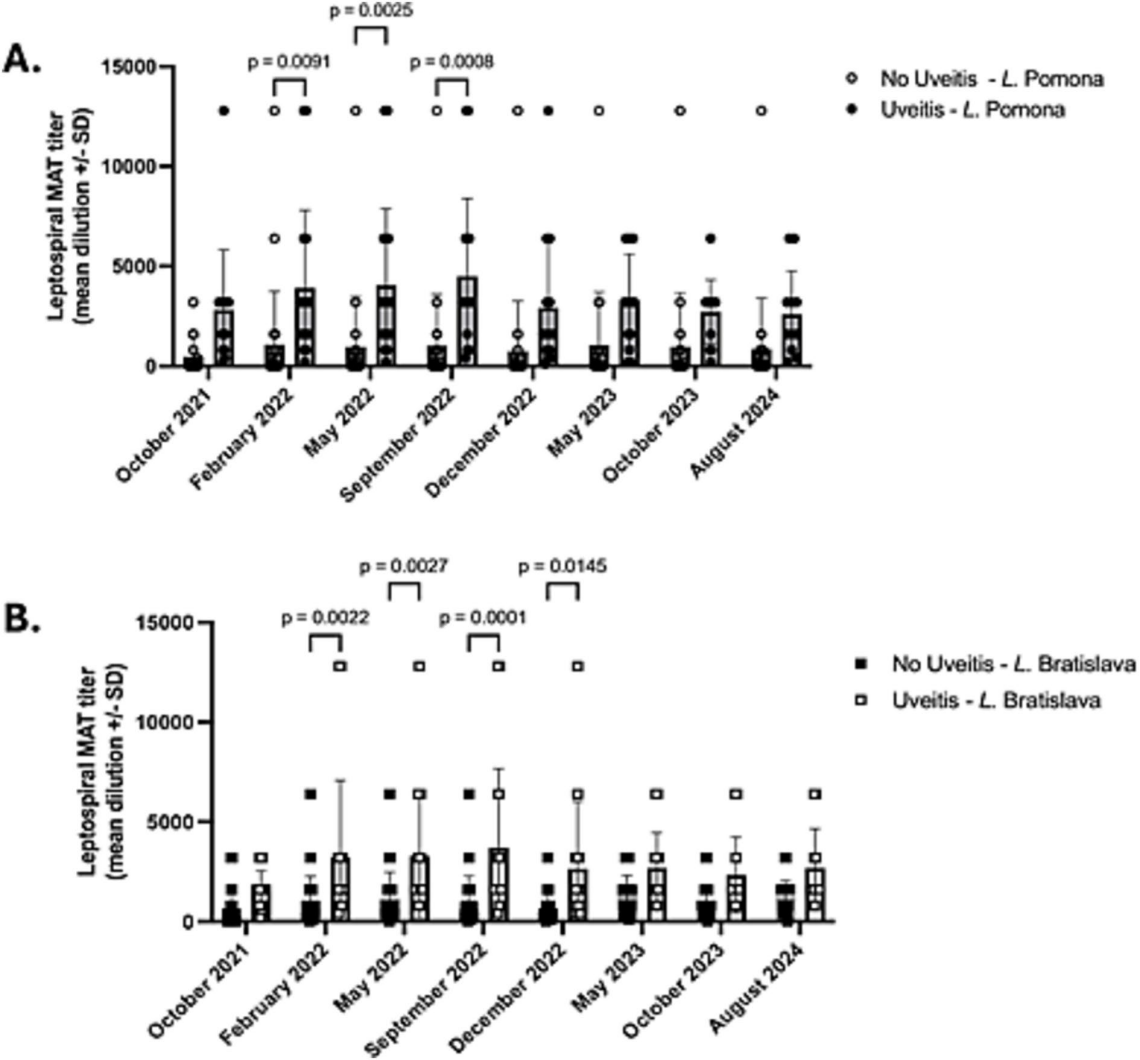
Mean and scatter plot distribution of micro-agglutination titers (MAT). **(A)** MAT *L.* pomona. Horses with uveitis had significantly higher mean titers than horses without uveitis on February 2022, May 2022, and September 2022, but there was no significant differences in titers on the other dates tests. **(B)** MAT *L.* Bratislava. Horses with uveitis had significantly higher mean titers than horses without uveitis in February 2022, May 2022, September 2022, and December 2022, but there were no significant differences in titers on the other dates. In animals with and without uveitis, there was no significant variation over time in *L.* Pomona (*p* = 0.984) or *L.* Bratislava (*p* = 0.1) serum titers throughout the study period.

In both uveitis and non-uveitis animals, serum titers of *L.* Grippotyphosa, Icterohemorrhagicae, Canicola, and Harjo were positive sporadically and, when detected, rarely higher than 1:400 (Supplementary Table S3). However, in horses with uveitis (*n* = 17), the *L.* Pomona and *L.* Bratislava serum titers were significantly elevated in February 2022, May 2022, and September 2022 compared to serum titers of horses, donkeys, and mules not developing uveitis (*p* < 0.022) (Table 2, Figure 3, Supplementary Table S3). Further, the *L.* Bratislava mean serum titers were significantly elevated in December 2022 compared to those of horses, donkeys, and mules not developing uveitis (*p* < 0.0145). On the other dates (October 2021, May 2023, October 2023, August 2024), there was no significant difference in mean titers of *L.* Pomona or *L.* Bratislava between animals that did and did not develop uveitis (*p* > 0.068). In animals with uveitis and without uveitis, the leptospiral titers remained elevated throughout the three-year follow-up period, with no significant differences in *L.* Pomona or *L.* Bratislava serum titers over time (*p* > 0.1) (Table 2; Figure 3).

**Table 2:** Mean (+/−SD) Leptospiral titers in horses with and without uveitis.

	October 2021	February 2022	May 2022	September 2022	December 2022	May 2023	October 2023	August 2024	Difference over time
Uveitis									
Pomona	3,738 +/−2,914	4,013 +/−4,850	3,964 +/−3,860	4,714 +/−3,944	2,921 +/−725	3,286 +/−2,316	2,732 +/−1,590	2,600 +/−2,163	*p* = 0.5832
Bratislava	1,587 +/−931	3,225 +/−3,845	3,294 +/−3,109	3,857 +/−4,066	2,739 +/−548	2,769 +/−1807	2,400 +/−375	2,691 +/−1968	*p* = 0.4953
No uveitis									
Pomona	769 +/−2,355	921 +/−2,472	841 +/−2,337	890+/−2,369	648 +/−487	960 +/−2,438	485 +/−765	860 +/−2,533	*p* = 0.9845
Bratislava	678 +/−1,334	959 +/−1,167	1,093 +/−1,231	771 +/−658	681 +/−355	1,362 +/−907	1,044 +/−250	1,252 +/−812	*p* = 0.1016
Uveitis vs no uveitis									
*p*-value Pomona	*p* = 0.0680	***p* = 0.0091**	***p*** **= 0.0025**	***p* = 0.0008**	*p* = 0.1301	*p* = 0.1115	*p* = 0.4228	*p* = 0.4860	
*P*-value Bratislava	*p* = 0.6871	***p* = 0.022**	***p* = 0.0027**	***p* = 0.0001**	***p* = 0.0145**	*p* = 0.2370	*p* = 0.3040	*p* = 0.2814	

*P-value: Difference between uveitis and no uveitis mean values for indicated serogroup. Mixed-effects analysis with Sidak’s multiple comparisons test. Significance was set as P ≤ 0.05.Pomona: L. Pomona, Bratislava: L. Bratislava. Bold values demonstrate statistical significance.*

**Figure 4:**
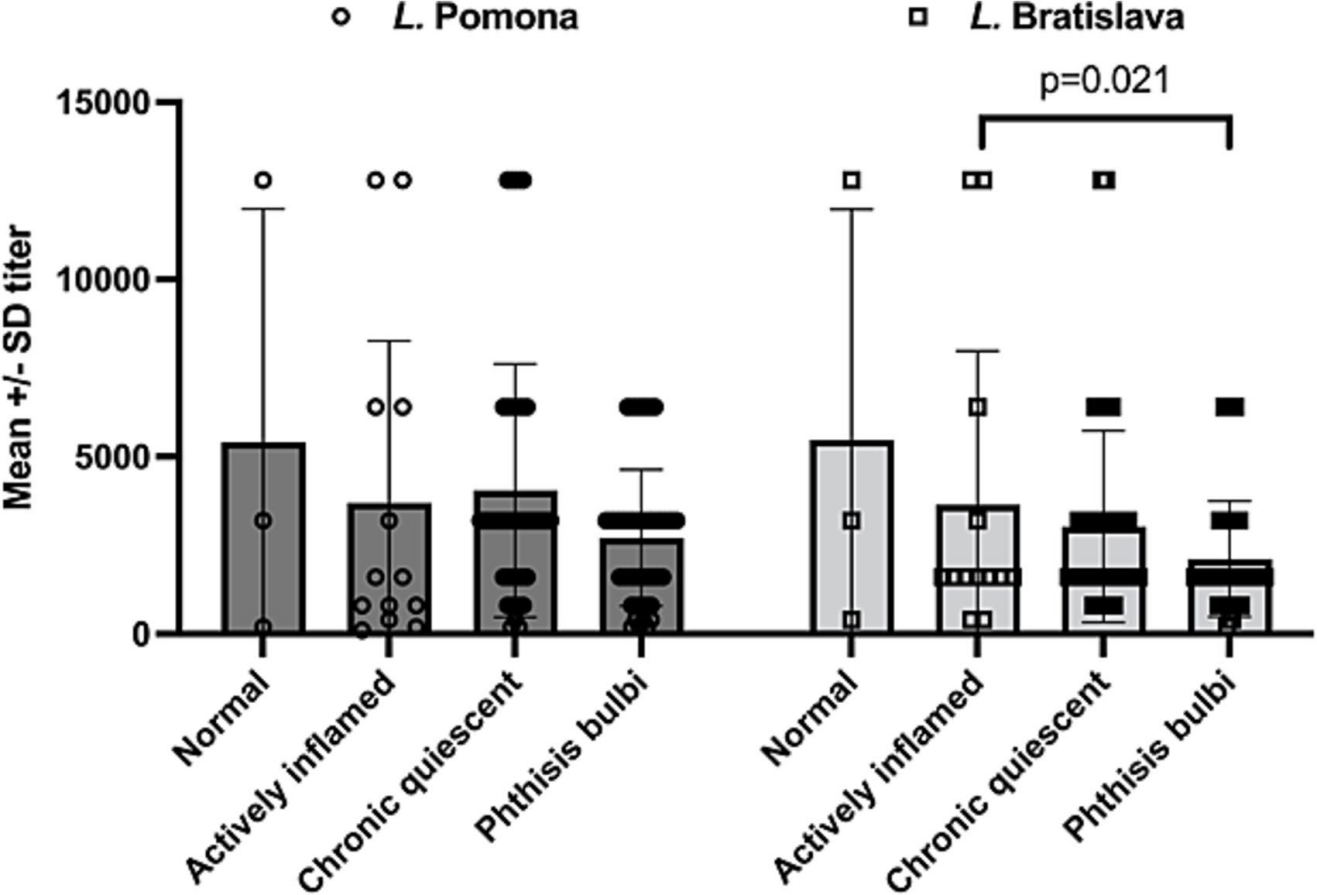
Serum leptospiral titers in horses with uveitis (*n* = 17). There was no significant difference in mean *L.* Pomona serum titers whether the eyes were normal (mean 1:5,400 +/−SD 6,581), actively inflamed (mean 1:3,684 +/−4,569), had chronic quiescent uveitis (blind or visual) (mean 1:4,231 +/−3,787), or had phthisis bulb (mean 1: 2,776 +/−1887) (*p* = 0.1432). However, MAT serum titers of *L.* Bratislava were significantly higher when the uveitis was active (mean 1:3,880 +/−5,519) compared to when the eyes had phthisis bulb (mean 1:1,996 +/−1,544). Each data point plotted represents a serum titer from an animal presenting with corresponding condition.

For the eyes that had uveitis (*n* = 17), there was no significant difference in mean *L.* Pomona serum titers (mean +/−SD) whether the eyes were normal (1:5,400 +/−SD 6581), actively inflamed (1:3,684 +/−SD 4,569), had chronic quiescent uveitis (blind or visual) (1:4,231 +/−SD 3787), or had phthisis bulb (1: 2,776 +/−SD 1,887) (*p* = 0.1432). However, serum titers (mean +/−SD) of *L.* Bratislava were significantly higher when the uveitis was active (1:3,880 +/−SD 5,519) compared to when the eyes had phthisis bulb (1:1,996 +/−SD 1,544) (*p* = 0.021) but no significant differences in mean titer were observed between when the eyes were quiescent chronic (blind or visual) (1: 3,221 +/−SD 2,848), normal (1: 5467 +/−SD 6503), or had active uveitis (*p* > 0.1721) (Figure 4).

Two subgroups existed based on their serum leptospiral titers in the 32 horses, donkeys, and mules that did not develop uveitis (in either eye) during the 34-month study period. One subgroup of 12 animals (11 horses, one donkey) had negative or low serum leptospiral titers (*L.* Pomona or *L.* Bratislava titers that were ≤ 1:800) throughout the study period (Supplementary Tables S1, S3). These low-titer, non-uveitis horses had a mean *L.* Pomona titer of 1:15.8 +/−SD 59.0 with a range in titers of 0 to 1:400, while the mean *L.* Bratislava titer was 1:338.2 +/− SD 256.1 with a range of 0 to 1:800. The second subset of 16 horses, three mules, and one donkey had high leptospiral titers (i.e., >1:800 at one or more time points) but did not develop signs of uveitis throughout the study period. These animals had a mean *L.* Pomona serum titer of 1:1,215.6 +/−SD 2,654.7 with a range of 0 to >1:12,800 and a mean *L.* Bratislava titer of 1:1,263.8 +/−SD 1,157.1 with a range of 0 to 1:6,400 (Supplementary Tables S1, S3). In animals (including horses, donkeys, and mules) without uveitis, there was no significant variation over time in *L.* Pomona (*p* = 0.984) or *L.* Bratislava (*p* = 0.1) serum titers throughout the study period (Table 2, Figure 3).

### 3.4 Water testing

**Figure 5:**
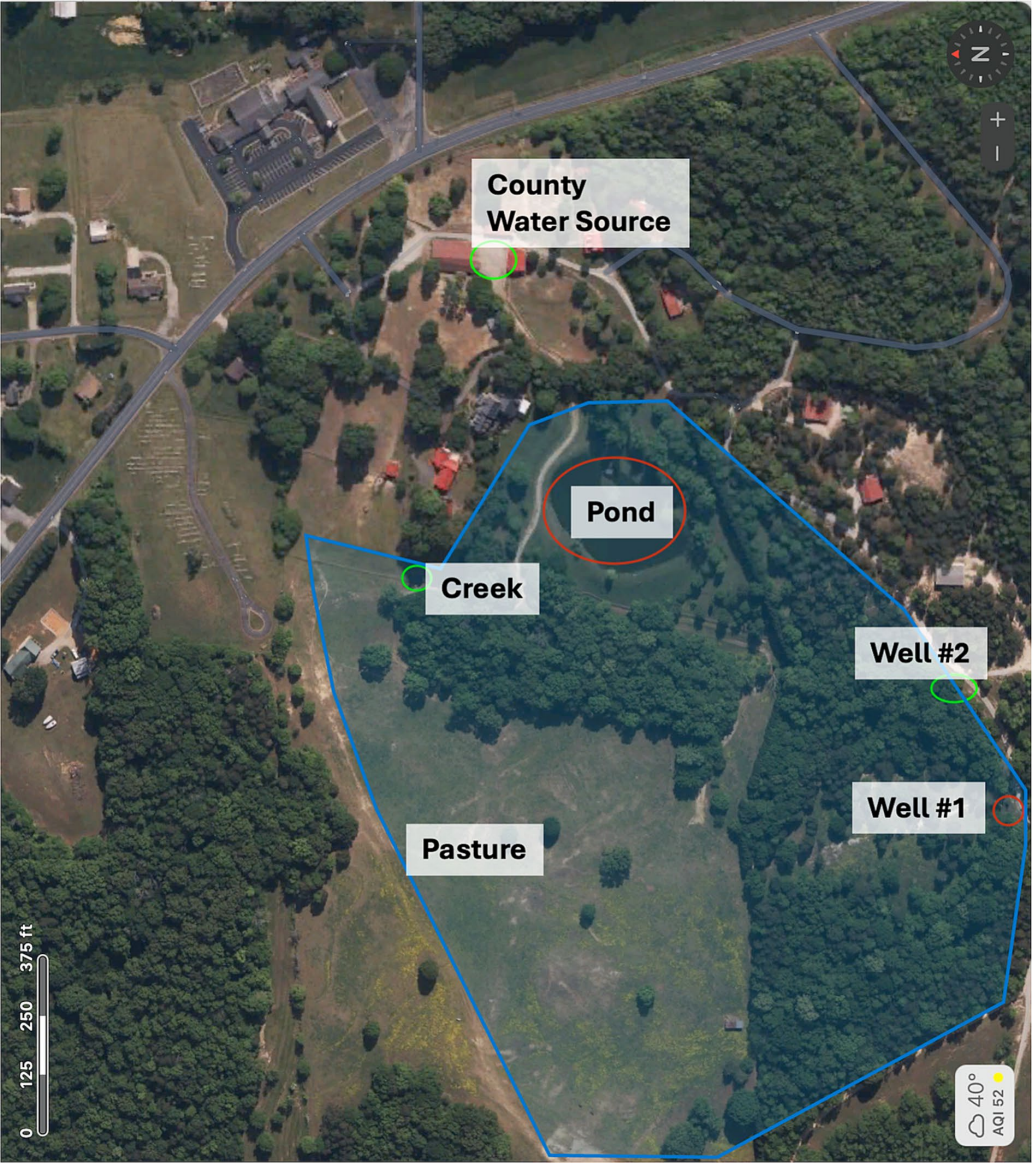
Location of water sources on farm. Five water sources the horses had access to were identified on the farm. This included the county water supply, a creek, a pond, and wells #1 and #2. In December 2022, the pond and well #2 were found to have high levels of leptospira, and in August 2024, these sources were also positive along with the creek and county water source.

Water sources accessible to all horses in the pasture were tested for Leptospiral content in December 2022 and August 2024 by a commercial environmental laboratory using quantitative PCR. Samples were collected from two separate water wells, a creek, a centrally located pond, and the county public water supply (Figure 5; Supplementary Table S4). In the December 2022 testing, leptospira was detected in well #1 (86 cell equivalents/100 mL) and the pond (593 cell equivalents/100 mL). At the same time, the other water sources were negative. In the August 2024 testing, the leptospiral levels were substantially higher than detected in December 2022, including positive leptospira in well #1 (2,871 cell equivalents/100 mL), the creek (64,699 cell equivalents/100 mL), the pond (127 cell equivalents/100 mL), and even the county water source (76 cell equivalents/100 mL). The other water source (Well #2) remained negative (Supplementary Table S4, Supplementary Figures S1, S2).

## 4 Discussion

Leptospira are bacterial organisms found in rivers, lakes, ponds, standing water, and sewage. Classification of leptospira species is complex and has changed in the past few years as genomic testing and sequencing for identifying organisms has become more common (28). Historically, serotyping, or antigenic classification into serovars, which relies on the use of specific monoclonal antibodies to identify exposure, led to the identification of more than 300 serovars within 30 serogroups (29). Serotyping is predominantly performed using the microscopic agglutination test (MAT), which evaluates the patient’s immune response and production of antibodies specific to the infecting leptospiral serogroup (30). The MAT test involves the incubation of serial dilutions of patient sera with a panel of live leptospiral organisms as antigens and reading the resulting agglutination under a darkfield microscope (31). However, the serovar classification of leptospira is a poor indicator of genetic relatedness as many serovars are shared by different species, whether or not they are pathogenic (28). For example, the serovar Pomona may represent several species, such as *L. interrogans* or *L. kirschneri* (28). Therefore, the use of a molecular genomic diagnosis is considered more specific and sensitive for the identification of disease-associated leptospira (29, 31).

Recently, genomic classifications of leptospira have been described (32), which is a separate classification system from the serovar antigenic classification (33). Based on molecular data, Leptospira species are now separated into two clades (P and S) and further subdivided into four subclades: P1 (high and low virulent subclades), P2 (intermediate virulent), S1 (saprophytic), and S2 (Saprophytic) (28, 32, 33). The P1 high virulent group includes *L. interrogans*, *L. kirschneri*, *L. noguchii, L. santarosai*, *L. mayottensis*, *L. borgpetersenii*, *L. alexanderi,* and *L. weilii*, which are the only known species associated with disease in animals. Future clinical diagnostic testing will use molecular genomic data to identify causative organisms and move away from MAT serology testing (33).

This study used MAT serology, which remains, for now, the gold standard for clinical testing of leptospira (29). However, MAT serology can only identify infecting Leptospira to the serogroup level (29, 34). Therefore, because only MAT serotyping was done and not molecular genetic typing, only serogroups were identified, not species or serovar. Therefore, serologic findings in this study are listed as *L.* Pomona, for example.

Leptospira can be classified if they cause “host-adapted” or “incidental” host infection (3, 4). Host-adapted strains are less likely to cause clinical disease and the host infection and shedding are prolonged. Conversely, incidental host leptospira are more likely to cause clinical disease and are associated with a marked serologic response (3). *Leptospira interrogans* serovar Pomona type kennewicki has been reported to be the most common pathogenic serovar for horses, followed by *Leptospira kirschneri* serovar Grippotyphosa (3). The principal wildlife host-adapted reservoirs for the serogroups associated with the equine disease include raccoons, skunks, opossums, foxes, deer (*L.* Pomona), and rodents (*L.* Grippotyphosa) (3). The organisms proliferate in the kidneys of adapted hosts and are shed in the urine, infecting other animals, such as horses, when they consume urine-contaminated water or food from an infected host-adapted species.

In this prospective cohort observational study, a large and diverse group of horses, mules, and donkeys on a farm were examined for over 34 months following an outbreak of suspected leptospirosis. Many of the animals were found to have elevated serum titers to *L.* Pomona and *L.* Bratislava. All animals were managed similarly on a single farm and pasture, experienced the same environmental factors, and had the same water sources and diet. This allowed a unique ability to study the natural development of disease in a closed herd of Equidae with few variables. We identified that following a suspected outbreak of leptospirosis, approximately one-third of the animals developed uveitis and blindness. Horses with uveitis had significantly elevated serum leptospiral titers compared to horses, mules, and donkeys that did not develop uveitis. Serum titers of *L.* Pomona were particularly high in horses with uveitis, as previously reported (1, 6, 8), but in these horses with uveitis, there were also significant titer elevations of *L.* Bratislava (see Figure 4). Although *L.* Bratislava was once thought to be host-adapted in the horse, recent studies suggest that it may not be host-adapted; instead, the elevation may be a cross-reaction (co-agglutination) with other serovars in the MAT due to widely shared genomic features among the serovars (35). Further, co-agglutination is very common in MAT reactions in the acute phase of the disease, and these cross-reacting strains decrease earlier (and faster) than the titers against the infectious strain (36). It was interesting to note, however, that the *L.* Bratislava titers were significantly elevated in horses during periods of active uveitis (compared to eyes with chronic uveitis, or phthisis bulbi), while the *L.* Pomona titers were not significantly different with the inflammatory state of the eyes (i.e., normal, acute uveitis, chronic uveitis, or phthisis bulbi) (see Figure 4). Was the elevated *L.* Bratislava serum titers secondary to overall increased inflammation or immune stimulation directly related to the pathogenesis of active uveitis or a result of MAT co-agglutination reactions? Additional studies, such as evaluating intraocular fluid titers, PCR detection, isolation, and typing of Leptospira strains recovered from intraocular fluids, may help determine its role in the pathogenesis of active uveitis.

Interestingly, the *L.* Grippotyphosa was not elevated in these horses from central North Carolina despite previous reports of horses from Eastern North Carolina having *L.* Grippotyphosa titers at an equal or higher rate than *L.* Pomona in horses with leptospiral-associated uveitis (1, 2). Furthermore, in the study reported herein, horses with uveitis had elevated *L.* Pomona and *L.* Bratislava titers that were constantly elevated without significant variation for over 34 months (see Table 2). Although serum leptospiral titers are elevated for a sustained period after natural infection, the elevated leptospiral serum titers observed in the horses of this study were prolonged and much longer than the 60-day duration of elevated titers reported after experimental infection of horses *with L.* Pomona (36). By 11 months after the initial examination, no horses developed additional uveitis (i.e., normal eye to active uveitis) despite maintaining high titers to leptospira. The water sources available to these horses had high and sustained levels of leptospira (substantially higher in the summer month [August 2024] compared to the December [2022] sampling—see Supplementary Table S4). Because horses were not restricted from contaminated water sources, it is possible that they were continuously re-exposed to leptospira, thus maintaining their elevated serum titers. Because no additional horses developed uveitis 18 months (after the 11-month examination) after the presumed initial infection, despite the continued leptospiral exposure, all the susceptible horses and eyes likely developed uveitis within these first 18 months, leaving no additional horses susceptible to developing additional cases of uveitis and blindness.

The signs of uveitis in the horses described in this report were chronic and severe, and they had rapid progression to phthisis bulbi, which commonly developed within 3 to 6 months after the observation of active uveitis. The most frequently observed clinical signs were posterior synechia, mature cataracts, and phthisis bulbi. Recurrent uveitis was relatively uncommon, observed in only nine eyes of eight horses (26% of 34 total eyes in 17 uveitis horses) over 3 years. One of these eyes had two bouts of uveitis, but no recurrence of inflammation was observed after the eyes developed phthisis bulbi. These clinical features associated with leptospiral-associated uveitis appear more severe and progress to blindness faster than other causes of recurrent uveitis, such as non-infectious immune-mediated causes (1, 24). Conversely, the rapid progression of uveitis in the horses in this report could be because they were not consistently treated.

Despite the same environmental leptospiral exposure as other horses, some horses had low leptospiral titers and did not develop uveitis. These horses may have a genetic resistance to developing leptospiral primary infections, have a more robust immune response to resist developing the disease, or were not exposed, i.e., did not drink from the contaminated water as other horses because of herd dynamics or other factors. Also, a subset of horses, mules, and donkeys developed high leptospiral serum titers without developing ocular disease. Some of these horses only had elevated *L.* Bratislava titers, which may be nonpathogenic in horses (35). However, 6 of 32 animals that did not develop uveitis had highly elevated *L.* Pomona serum titers, with one horse having an *L.* Pomona serum titer persistently higher than 1:6,400 and another horse having consistent serum titers of greater than 1:12,500 (see Supplementary Table S2). It is possible that these six horses with the elevated *L.* Pomona serum titers had subclinical uveitis; however, this is unlikely because chronic ocular manifestations of the disease were never observed during nearly 3 years of follow-up. Does a genetic predisposition protect these horses from developing uveitis following leptospiral exposure? It is possible that specific genetic haplotypes do not exhibit the autoantigens in the eye that are recognized by T-cells activated by leptospiral infections; thus, these horses do not develop uveitis (11, 13). Further work is warranted to determine genetic susceptibility to leptospiral infection, the genomic influence on immunopathogenesis in uveitis, and the role of genetics on the development of uveitis in horses.

All donkeys (*n* = 2), mules (*n* = 3), and miniature horses (*n* = 5) at this farm did not develop uveitis despite several (1 donkey, three mules, and four miniature horses) developing high serum leptospiral titers (See Supplementary Table S3). There is widespread evidence that donkeys and mules are susceptible to and generate antibody titers to leptospira (37–39). However, it has been suggested that donkeys have a lower incidence of recurrent uveitis than horses (40). In a study of 207 donkeys in the UK, uveitis was found to be relatively uncommon, with only approximately 3% (*n* = 6/207) of animals having signs of uveitis (41). Early publications state that uveitis is common in mules (25, 42), but actual reports of mules with uveitis are rare. In one study, only one mule in 133 cases (0.75%) had recurrent uveitis (43). It is possible that in donkeys and mules, leptospiral antigen-activated T cells do not react to ocular auto-antigens, thus reducing the chances of an immunological reaction and development of uveitis, as commonly occurs in horses (10, 11). Additionally, uveitis has rarely been reported in miniature horses, with only two eyes showing signs of uveitis in 45 Caspian miniature horses (44) and no signs of uveitis in a survey of 53 American miniature horses (45). Further genetic and immunologic analysis is warranted to determine why donkeys, mules, and miniature horses appear less susceptible to leptospirosis and the development of uveitis.

### 4.1 Limitations of the study

The ocular health and visual status of the horses on this farm were unknown before the outbreak of leptospira. Therefore, we cannot determine if a pre-existing disease or elevated leptospiral titers were present. However, the farm owner did not observe any horse with ocular discomfort or blindness before January 2021. Ocular examinations throughout the study were limited to every 3–11 months, which did not permit detailed information on timing of onset of disease and the disease progression rate. Furthermore, advanced ocular examination techniques, such as ultrasonic imaging, were not done, limiting the complete characterization of the ocular disease. Additionally, diagnostics such as aqueous humor leptospiral titers, calculation of C values, PCR, or molecular identification were not done, which is recommended for definitive diagnosis of leptospiral-associated uveitis (31–33). Finally, diagnostic tests for other infectious diseases associated with uveitis, such as borreliosis, were not performed because of a lack of clinical signs or history of tick exposure (27). However, no clinical signs were observed in this group of horses supporting other systemic diseases or other differential diagnoses, so further testing was not recommended (46).

## 5 Conclusion

Approximately one-third of horses on a farm exposed to leptospira developed uveitis and blindness. *L.* Pomona and *L.* Bratislava were significantly elevated in horses with uveitis, and both serogroups had elevated titers for over 3 years after the suspected primary infection. Disease progression was rapid, leading to blindness in many horses. Observed recurrences of uveitis were uncommon. Furthermore, donkeys, mules, and miniature horses did not develop ocular disease on this farm. These data indicate that further research is warranted to investigate the genetic and immunological aspects of the pathogenesis and susceptibility of leptospiral-associated uveitis.

High levels of leptospiral DNA found in water samples on this farm indicate that contaminated water sources, particularly one specific well and a pond, may be the source of leptospiral infection on this farm. Restricting the horses on this farm from the contaminated water sources may have prevented further disease. Further study is needed to determine effective measures to avoid exposure of horses to leptospira and disease development.

## Data Availability

The original contributions presented in the study are included in the article/Supplementary material, further inquiries can be directed to the corresponding author.

## Footnotes

^1^ https://www.vet.cornell.edu/animal-health-diagnostic-center
