# Supplementary material for: Uveitis and blindness in a closed herd of Equidae following leptospiral infection
Source: Front Vet Sci. 2025 Jan 6;11:1504990. doi: 10.3389/fvets.2024.1504990 (PMC11743373; doi:10.3389/fvets.2024.1504990)
Supplement: Supplementary file 1 [file Data_Sheet_1.DOCX]

**Prevalence of uveitis and blindness in a closed herd of equidae following leptospiral infection**

Gerras J,^1^ Young K,^1^ Roberts D, ^1^ Waldman G,^2^ Salmon JH,^1^ Gilger BC^1^

1. Department of Clinical Sciences, North Carolina State University, Raleigh, NC 27607

2. Rivendell Mobile Large Animal, Advance, NC 27006

**Supplemental materials**

**S1 Table. Horse population summary**

| **Horses without uveitis** | | | | | |
| --- | --- | --- | --- | --- | --- |
| ***Negative or low serum lepto titers (n=11 horses, 1 donkey; 3 deceased; 9 alive)*** | | | | | |
| **Horse name** | **Breed** | **Age (yrs)** | **Gender** | **Coat color** | **Uni-or bilateral** |
| Bandit | Quarter horse | 20 | MC | White/black paint | - |
| Blaze | Quarter horse | 20 | M | Dun | - |
| Blondie | Mini horse | 10 | F | Bay | - |
| Bojangles | Tennessee WH | 9 | MC | Bay | - |
| Dusty (Deceased) | Quarter horse | 22 | MC | Buckskin | - |
| Jellybean (Deceased) | Pony | 20 | F | Paint | - |
| Joseph | Thoroughbred | 18 | MC | Bay | - |
| Little lady | Quarter horse | 22 | F | Bay | - |
| Pebbles | Quarter horse | 20 | F | Chestnut | - |
| Tillie | Thoroughbred | 8 | F | Bay | - |
| Leroy (Deceased) | TWH | 23 | M | Black | - |
| Arvid | Donkey | 25 | MC | Dun | - |
| ***Medium or high lepto titers (n=16 horses, 3 mules, 1 donkey; 2 deceased; 18 alive)*** | | | | | |
| Big Buddy (Deceased) | Draft cross | 29 | MC | Chestnut | - |
| Bouncer James | Mini horse | 26 | MC | Dun | - |
| Doolittle | Mini horse | 20 | MC | Black | - |
| Dori | Quarter horse | 26 | F | Bay | - |
| Lego | Haflinger | 15 | MC | Chestnut | - |
| Lily | Hackney pony | 18 | MC | Chestnut | - |
| Oreo | Percheron | 12 | MC | Black/white paint | - |
| Peanut | Pony | 25 | F | Chestnut | - |
| Rowdy | Mini | 15 | MC | White/chestnut | - |
| Roxie (Deceased) | Quarter horse | 20 | F | Gray | - |
| Sally | Pony | 15 | F | Chestnut | - |
| Ella | Quarter horse | 23 | F | Chestnut | - |
| Neytiri | New Forest pony | 11 | F | Bay | - |
| Penny | Pony | 22 | F | Dun | - |
| Little buddy | Hackney pony | 21 | MC | Bay | - |
| Cody | Mini horse | 20 | MC | Chestnut | - |
| Dolly | Donkey | 7 | MC | Dun | - |
| Magic | Mule | 10 | MC | Black | - |
| Ultra | Mule | 4 | F | Bay | - |
| Birdie | Mule | 12 | F | Paint | - |
| **Horses with uveitis** | | | | | |
| ***Medium or high lepto titers* (n=17 horses, 3 deceased, 14 alive)** | | | | | **Uni-or bilateral** |
| Camilla | Quarter horse | 18 | F | Grulla | bilateral |
| Carrie | Quarter horse | 21 | F | Chestnut | Bilateral |
| Chance | Quarter horse | 18 | MC | Black | Bilateral |
| Cobalt | New Forest pony | 13 | MC | Bay | Bilateral |
| Jim Dandy (deceased) | Quarter horse | 21 | MC | Chestnut | Bilateral |
| Judd | Quarter horse | 14 | MC | Chestnut | Bilateral |
| Lacy | Appaloosa | 22 | F | White | Bilateral |
| Red | Gypsy vanner | 13 | MC | White/red roan | Bilateral |
| Rosen | Fjord | 25 | F | Dun | Bilateral |
| Slick | Draft cross | 25 | F | White/chestnut | Bilateral |
| Sundancer | Mustang | 20 | F | Dun | Unilateral |
| Trenche | Fjord | 23 | F | Dun | Bilateral |
| Delila | Percheron | 21 | F | Black | Bilateral |
| Dupont (deceased) | Percheron cross | 18 | MC | Black/white | Bilateral |
| Hannah | Welsh pony | 13 | F | Bay | Unilateral |
| Meara (deceased) | Thoroughbred | 20 | F | Chestnut | Bilateral |
| Mo | Draft cross | 25 | MC | Bay | Bilateral |

OS – left eye; OD – right eye; OU-both eyes

F – female, MC – male castrated

| **S2 Table.** Clinical features of horses that developed uveitis over the course of 3 years | | | | | | | | |
| --- | --- | --- | --- | --- | --- | --- | --- | --- |
| **Horse #** | **Oct 2021**  (Month 0) | **Feb 2022**  (Month 4) | **May 2022**  (Month 7) | **Sept 2022**  (Month 11) | **Dec 2022**  (Month 14) | **May 2023**  (Month 19) | **Oct 2023**  (Month 24) | **August 2024**  (Month 34) |
| 1. Camilla | Blind OS  Chronic uveitis OD | Phthisis OS  Blind OD | Phthisis OU | Phthisis OU | Phthisis OU | Phthisis OU | Phthisis OU | Phthisis OU |
| 2. Carrie | Normal OU | Normal OU | Blind OU | Blind OU | Phthisis OS  **Active OD** | Phthisis OU | Phthisis OU | Phthisis OU |
| 3. Chance | Blind OU | Blind OU | Blind OU | Blind OU | Phthisis OU | Phthisis OU | Phthisis OU | Phthisis OU |
| 4. Cobalt | Blind OS  Chronic uveitis OD | Blind OS  Chronic uveitis OD | Blind OS  **Active OD** | Blind OS  **Active OD** | Phthisis OU | Phthisis OU | Phthisis OU | Phthisis OU |
| 5. Delilah | *Not examined* | *Not examined* | Phthisis OU | Phthisis OU | Phthisis OU | Phthisis OU | Phthisis OU | Phthisis OU |
| 6. Dupont | Blind OS  Normal OD | Blind OS  **Active OD** | Phthisis OU | *Died* | - | - | - | - |
| 7. Hannah | Normal OU | **Active OS**  Normal OD | Chronic uveitis OS  Normal OD | Blind OS  Normal OD | **Active OS**  Normal OD | Blind OS  Normal OD | Phthisis OS  Normal OD | Phthisis OS  Normal OD |
| 8. Jim Dandy | Chronic uveitis OU | Phthisis OD  Chronic uveitis OS | Phthisis OD  Chronic uveitis OS | *Died* | - | - | - | - |
| 9. Judd | Chronic uveitis OU | Chronic uveitis OS  **Active OD** | Chronic uveitis OS  Chronic uveitis OD | Chronic uveitis OS  Chronic uveitis OD | Phthisis OS  Chronic uveitis OD | Phthisis OS  Chronic uveitis OD | Phthisis OS  Chronic uveitis OD | Phthisis OS  Chronic uveitis OD |
| 10. Lacy | Chronic uveitis OD  Blind OS | Phthisis OU | Phthisis OU | Phthisis OU | Phthisis OU | Phthisis OU | Phthisis OU | Phthisis OU |
| 11. Meara | Chronic uveitis OD  Blind OD | Chronic uveitis OD  Blind OD | Blind OU | *Died* | - | - | - | - |
| 12. Mo | Phthisis OU | Phthisis OU | Phthisis OU | Phthisis OU | Phthisis OU | Phthisis OU | Phthisis OU | Phthisis OU |
| 13. Red | Normal OS  Chronic uveitis OD | Normal OS  Chronic uveitis OD | Chronic uveitis OS  Blind OD | Chronic uveitis OS  Blind OD | Chronic uveitis OS  Phthisis OD | **Active**  **uveitis OS**  Phthisis OD | Phthisis OU | Phthisis OU |
| 14. Rosen | Chronic uveitis OS  Normal OD | Chronic uveitis OS  Normal OD | Chronic uveitis OS  Normal OD | **Active**  **uveitis OU** | **Active**  **uveitis OU** | **Active**  **uveitis OU** | **Active**  **uveitis OU** | Phthisis OU |
| 15. Slick | Blind OS  Normal OD | Blind OS  Phthisis OD | Blind OS  Phthisis OD | Blind OS  Phthisis OD | **Active OS**  Phthisis OD | Phthisis OU | Phthisis OU | Phthisis OU |
| 16. Sundancer | Chronic uveitis OS  Normal OD | Chronic uveitis OS  Normal OD | Chronic uveitis OS  Normal OD | Chronic uveitis OS  Normal OD | Chronic uveitis OS  Normal OD | Chronic uveitis OS  Normal OD | Chronic uveitis OS  Normal OD | Chronic uveitis OS  Normal OD |
| 17. Trenche | Phthisis OU | Phthisis OU | Phthisis OU | Phthisis OU | Phthisis OU | Phthisis OU | Phthisis OU | Phthisis OU |

OS – left eye

OD – right eye

OU – both eyes

Normal – eye did not have signs of active or chronic uveitis

Active – eye had signs of active uveitis

Chronic uveitis – eye had signs of quiescent chronic uveitis, but visual (Quiescent / chronic uveitis (Visual)

Blind – eye had signs of quiescent chronic uveitis but not visual (Quiescent / chronic uveitis (Blind)

Phthisis – eye had phthisis bulbi

**S3 Table. Serum leptospiral titers**

| **Horses without uveitis** | | | | | | |  | |  |
| --- | --- | --- | --- | --- | --- | --- | --- | --- | --- |
| ***Negative or low serum leptospiral titers (n=11 horses, 1 donkey; 3 deceased; 9 alive)***  ***Serum leptospiral titers*** | | | | | | |  | |  |
| **Horse #** | **October 2021** | **February 2022** | **May 2022** | **September 2022** | **December 2022** | **May 2023** | **October 2023** | | **August 2024** |
| 1. Bandit | Negative | Ictero 1:100 | Bratis 1:400 Icter 1:100 | Ictero 1:100 | Negative | Ictero 1:100 Bratis 1:100 | Negative | | Ictero 1:100 |
| 2. Blaze | 1:400 bratis | Grippo 1:200 Bratis 1:800 | Bratis 1:400 Grippo 1:200 Ictero 1:400 | Bratis 1:400 Grippo 1:100 | Bratis 1:800 Grippo 1:200 Pom 1:100 | Grippo 1:200 Bratis 1:800 | Grippo 1:200  Bratis 1:800 | | Pom 1:100  Can 1:200 Ictero 1:100, Grippo 1:200  Bratis 1:800 |
| 3. Blondie | Bratis 1:200 | Bratis 1:400 | Bratis 1:800 | Bratis 1:200 | Bratis 1:400 | Bratis 1:400 | Bratis 1:800 | | Pom 1:800 Can 1:400  Ictero 1:100 Harjo 1:100  Bratis:1600 |
| 4. Bojangles | Ictero 1:100 Bratis 1:100 | Ictero 1:200 Bratis 1:400 | Bratisa 1:800 Grippo 1:200 Harjo 1:100 | Bratis 1:200 Hardjo 1:100 Ictero 1:100 | Bratis 1:200 | Ictero 1:200 Hardjo 1:200 Bratis 1:400 | Ictero 1:100  Hardjo 1:100 Bratis 1:400 | | Can 1:200  Ictero 1:400 Grippo 1:100 Harjo 1:200 Bratis 1:400 |
| 5.Dusty (Deceased) | Bratis 1:100 | Bratis 1:400 | Bratis 1:200 | Bratis 1:100 | Bratis 1:200 | - | - | | - |
| 6. Jelly bean (Deceased) | Bratis 1:100 | Bratis 1:400 | Ictero 1:100 Bratis 1:400 | Ictero 1:100 Grippo 1:100 Bratis 1:400 | Ictero 1:100 Bratis 1:400 | Ictero 1:100 Grippo 1:100 Bratis 1:800 | - | | - |
| 7. Joseph | Negative | Pom 1:200 Bratis 1:400 | Bratis 1:400 | Bratis 1:400 | Bratis 1:200 | Bratis 1:800 | Bratis 1:800 | | Bratis 1:400 |
| 8.Little lady | Negative | Pom 1:100 Bratis 1:200 | Bratis 1:200 | Bratis 1:100 | Bratis 1:100 | Bratis 1:400 | Bratis 1:200 | | Bratis 1:400 |
| 9.Pebbles | Negative | Bratis 1:400 | Bratis 1:800 | Bratis 1:200 | Bratis 1:100 | Bratis 1:800 | Bratis 1:400 | | Bratis 1:800 |
| 10. Tillie | Bratis 1:200 | Pom 1:400 Ictero 1:100 Bratis 1:400 | Pom 1:100 Bratis 1:400 | Pom 1:100 Bratis 1:400 | Bratis 1:400 | Pom 1:100 Ictero 1:100 Bratis 1:800 | Bratis 1:800 | | Icterio 1:100  Bratis 1:800 |
| 11. Leroy (Deceased) | Ictero 1:100 | Pom 1:200 Ictero 1:400 Grippo 1:100 Bratis 1:400 | Pom 1:100 Ictero 1:400 Grippo 1:100 Bratis 1:400 | - | - | - | - | | - |
| 12.. Arvid (Deceased) donkey | Negative | Bratis 1:200 | Bratis 1:400 Ictero 1:100 | Bratis 1:200 Ictero 1:100 | Bratis 1:100 | - | - | | - |
|  |  |  |  |  |  |  |  | |  |
| ***Medium or high leptospiral titers (n=16 horses, 3 mules, 1 donkey; 2 deceased; 18 alive)*** | | | | | | |  | |  |
| 1.Bouncer James mini | Pom 1:1600  Ictero 1:200  Bratis 1:800 | Pom 1:1600 Ictero 1:200 Bratis1:800 | Pom 1:1600 Ictero 1:400 Bratis 1:1600 | Pom 1:1600 Ictero1:200 Bratis 1:800 | Pom 1:800 Ictero 1:100 Batis 1:800 | Pom 1:3200 Ictero 1:200 Hardjo 1:200 Bratis 1:1600 | Pomo 1:1600 Ictero 1:200 Bratis 1:800 | | Pom 1:800 Can 1:200  Ictero 1:400  Grippo 1:100  Bratis 1:1600 |
| 2. Big Buddy (Deceased) | Bratis 1:200 | Bratis 1:800 | Bratis 1:1600 Ictero 1:100 | Bratis 1:400 Ictero 1:100 | Bratis 1:800 | Bratis 1:800 | - | | - |
| 3. Doolittle mini | Ictero 1:100 Bratis 1:800 | Ictero 1:400 Bratis 1:1600 | Pom 1:100 Ictero 1:400 Bratis 1:1600 | Pom 1:200 Ictero 1:200 Bratis 1:1600 | Ictero 1:200 Bratis 1:1600 | Pom 1:200 Ictero 1:400 Hardjo 1:100 Bratis 1:1600 | Pom 1:100 Ictero 1:200 Bratis 1:1600 | | Can 1:100  Ictero 1:100  Grippo 1:100  Bratis 1:800 |
| 4. Dori | Negative | Bratis 1:1600 | Bratis 1:800 | Ictero 1:100 Grippo 1:100 Bratis 1:800 | Bratis 1:400 | Bratis 1:1600 | Bratis 1:800 | | Can 1;100  Icterio 1:100  Grippo 1:100 Bratis 1:800 |
| 5. Lego | Pom 1:800 | Pom 1:800 Bratis 1:200 | Pom 1:400 Bratis 1:200 | Pom 1:800 Bratis 1:1600 | Pom 1:400 Bratis 1:100 | Pom 1:800 Bratis 1:400 | Pom 1:800 Bratis 1:200 | | Pom 1:800  Bratis 1:400 |
| 6. Lily | Ictero 1:100 Bratis 1:800 | Pom 1:400 Can 1:100 Ictero 1:200 Bratis 1:800 | Pom 1:400 Can 1:100 Ictero 1:200 Bratis 1:800 | Pom 1:400 Ictero 1:200 Bratis 1:1600 | Pom 1:200 Ictero 1:200 Bratis 1:400 | Pom 1:400 Cani 1:100 Ictero 1:200 Bratis 1:800 | Pom 1:200 Can 1:100 Ictero 1:200 Bratis 1:800 | | Pom 1:200 Can 1:400  Ictero 1:200  Brat 1:800 |
| 7. Oreo | Pom 1:3200  Bratis 1:800 | Pom 1:6400 Ictero 1:200 Bratis 1:1600 | Pom 1:3200 Ictero 1:100 Hardjo 1:100 Bratis 1:1600 | Pom 1:3200 Ictero 1:100 Bratis 1:1600 | Pom 1:1600 Ictero 1:100 Bratis 1:800 | Pom 1:3200 Ictero 1:100 Bratis 1:1600 | Pom 1:3200 Ictero 1:100 Bratis 1:1600 | | Pom 1:1600  Can 1:100  ictero 1:100  Bratis 1:1600 |
| 8. Peanut | Negative | Pom 1:1600 Can 1:100 Ictero 1:100 Grippo 1:200  Hardjo 1:100 Bratis 1:1600 | Pom 1:800 Can 1:100 Ictero 1:100 Grippo 1:200 Hardjo 1:100 Bratis 1:800 | Pomo 1:800 Ictero 1:100 Grippo 1:200 Bratis 1:400 | Pom 1:400 Grippo 1:100 Hardjo 1:100 Bratis 1:200 | Pom 1:400 Grippo 1:100 Hardjo 1:100 Bratis 1:1600 | Pom 1:200 Grippo 1:100 Bratis 1:400 | | Pom 1:400 Can 1:100 Grippo 1:100 Bratis 1:800 |
| 9. Rowdy mini | Pom 1:3200 Ictero 1:400 Bratis 1:1600 | Pom 1:1600 Can 1:100 Ictero 1:800 Bratis 1:1600 | Pom 1:3200 Can 1:100 Ictero 1:800 Grippo 1:100 Bratis 1:3200 | Pomona 1:3200  Ictero 1:800 Bratis 1:1600 | Pomona 1:1600  Ictero 1:400 Bratis 1:1600 | Pom 1:3200 Ictero 1:800 Hardjo 1:100 Bratis 1:3200 | Pom 1:1600 Ictero 1:400  Bratis 1:1600 | | Pom 1:1600 Can 1:200 Ictero 1:800 Bratis 1:3200 |
| 10. Roxie (Deceased) | Pom 1:1600 Can 1:100 Ictero 1:400 Bratis 1:400 | Pom 1:1600 Can 1:200 Ictero 1:400 Bratis 1:800 | Pom 1:1600 Can 1:100 Ictero 1:400 Bratis 1:800 | Pom 1:1600 Can 1:100 Ictero 1:400 Bratis 1:800 | Pom 1:800 Can 1:100 Ictero 1:200 Bratis 1:400 | - | - | | - |
| 11.Sally | Pom 1:800  Grippo 1:100 Bratis 1:3200 | Pom 1:800 Ictero 1:100 Grippo 1:100  Bratis 1:3200 | Pom 1:800 Ictero 1:100 Grippo 1:200 Bratis 1:3200 | Pom 1:800 Ictero 1:100 Grippo 1:200 Bratis 1:3200 | Pom 1:400 Grippo 1:100 Bratis 1:3200 | Pom 1:800 Ictero 1:100 Hardjo 1:100 Bratis 1:3200 | Pom 1:400  Grippo 1:100 Bratis 1:3200 | | Pom 1:400  Ictero 1:100  Grippo 1:200  Bratis 1:1600 |
| 12. Ella | Bratis 1:800 | Bratis 1:800 | - | Ictero1:100 Bratis 1:800 | Bratis 1:800 | Ictero 1:100 Hardjo 1:100 Bratis 1:3200 | Ictero 1:100  Hardjo 1:100  Bratis 1:1600 | | Can 1:400, Ictero 1:100 Grippo 1:100  Harjo 1:100, Bratis 1:1600 |
| 13. Neytiri | Pom 1:1600 Can 1:200 Bratis 1:3200 | Pom 1:12,800 Ictero 1:3200 Grippo 1:100  Hardjo 1:200 Bratis 1:6400 | Pom 1:12,800 Ictero 1:1600 Grippo 1:100 Hardjo 1:200 Bratis 1:6400 | Pom >1:12,800 Ictero 1:1600 Grippo 1:100 Hardjo 1:100 Bratis 1:6400 | Pom 1:12,800 Ictero 1:800 Hardjo 1:100 Bratis 1:1600 | Pom 1:12,800 Ictero 1:800 Hardjo 1:100 Bratis 1:3200 | Pom 1:12,800 Ictero 1:800 Harjo 1:100 Bratis 1:3200 | | Pom 1:12,800  Ictero 1:1600 Harjo 1:100  Bratis 1:3200 |
| 14. Penny | Ictero 1:100 Bratis 1:400 | Pom 1:400 Ictero 1:100 Bratis 1:800 | Pom 1:200 Ictero 1:200 Bratis 1:800 | Pom 1:200 Ictero 1:200 Bratis 1:800 | Pom 1:100 Ictero 1:100 Bratis 1:800 | Pom 1:200 Ictero 1:200 Bratis 1:1600 | Pom 1:100 Ictero 1:100 Bratis 1:800 | | Pom 1:100  Can 1:100  Ictero 1:200 Bratis 1:1600 |
| 15. Little buddy | Pom 1:400 Ictero 1:200 Bratis 1:800 | Pom 1:400 Ictero 1:400 Bratis 1:800 | Pom 1:800 Ictero 1:400 harjo 1:100 Bratis 1:1600 | Pom 1:800 Ictero 1:400 Bratis 1:800 | Pom 1:400 Ictero 1:200 Bratis 1:800 | Pom 1:800 Ictero 1:400 Hardjo 1:100 Bratis 1:1600 | Pom 1:800 Ictero 1:400 Bratis 1:800 | | Pom 1:800 Can 1:200 Ictero 1:400 Grippo 1:100, Bratis 1:1600 |
| 16. Cody mini | Bratis 1:400 | Ictero 1:100 Bratis 1:800 | Ictero 1:100 Bratis 1:800 | Ictero 1:100 Bratis 1:800 | Bratis 1:800 | Bratis 1:1600 | Bratis 1:800 | | Ictero 1:100 Bratis 1:1600 |
| 17. Dolly | Bratis 1:100 | Bratis 1:800 | Bratis 1:800 | Bratis 1:200 | Bratis 1:400 | Bratis 1:1600 | Bratis 1:800 | | Bratis 1:800 |
| 18. Magic | Pom 1:200 Ictero 1:200 Bratis 1:800 | Pom 1:200 Ictero 1:200 Bratis 1:800 | Pom 1:200 Ictero 1:200 Bratis 1:800 | Pom 1:200 Ictero 1:200 Grippo1:100 Bratis 1:800 | Pom 1:200 Ictero 1:200 Bratis 1:400 | Pom 1:200 Ictero 1:200 Grippo 1:100 Bratis 1:1600 | Pom 1:200 Ictero 1:200 Bratis 1:800 | | Pom 1:200 Can 1:200; Ictero 1:200  Bratis 1:800 |
| 19. Ultra mule | Negative | Pom 1:100 Bratis 1:800 | Pom 1:100 Hardjo 1:100 Bratis 1:800 | Pomona 1:100 Bratis 1:800 | Bratis 1:400 | Pom 1:100 Bratis 1:1600 | Pom 1:100 Harjo 1:100 L. Bratis 1:800 | | Pom 1:100 Harjo 1:100  Bratis 1:1600 |
| 20. Birdie mule | Pom 1:200 Bratis 1:400 | Pom 1:400 Bratis 1:800 | Pom 1:400 Bratis 1:1600 | Pom 1:800 Bratis 1:800 Ictero1:100 Hardjo 1:100 | Pom 1:400 Bratis 1:400 | Pom 1:800 Hardjo 1:200 Bratis 1:1600 | Pom 1:800 L. hardjo 1:100 Bratis 1:1600 | | Pom 1:800  Can 1:400  Ictero 1:100  Harjo 1:100  Bratis 1:1600 |
|  |  |  |  |  |  |  |  | |  |
| **Horses with uveitis** | | | | | | |  | |  |
| ***Medium or high lepto titers* (n=17 horses, 3 deceased, 14 alive)** | | | | | | | | | |
| **Horse #** | **October 2021** | **February 2022** | **May 2022** | **September 2022** | **December 2022** | **May 2023** | | **October 2023** | **August 2024** |
| 1. Camilla | Pom 1:3200 Ictero 1:200 Bratis 1:1600 | Pom 1:1600 Ictero 1:400 Grippo 1:100 Bratis 1:1600 | Pom 1:3200 Ictero 1:400 Grippo 1:200 Bratis 1:1600 | Pom 1:6400 Ictero 1:400 Grippo 1:100 Bratis 1:1600 | Pom 1:1600 Ictero 1:200 Grippo 1:100 Bratis 1:800 | Pom 1:3200 Ictero 1:400 Grippo 1:100 Bratis 1:1600 | Pom 1:3200 L. Ictero 1:200 Grippo 1:100 Bratis 1:1600 | | Pom 1:6400; Ictero 1:400, Grippo 1:200, Bratis 1:1600 |
| 2. Carrie | Pom 1:3200 Ictero 1:800 Bratis 1:3200 | Pom 1:12,800 Ictero 1:1600 Grippo 1:100 Bratis 1:12,800 | Pom 1:12,800 Ictero 1:800 Grippo 1:200 Bratis 1:6400 | Pom 1:12,800 Ictero 1:800 Grippo 1:200 Bratis 1:12,800 Hardjo 1:100 | Pom 1:12,800 Ictero 1:400 Grippo 1:100 Bratis 1:6400 | Pom 1:6400 Ictero 1:800 Grippo 1:100 Hardjo 1:100 Bratis 1:3200 | Pom 1:6400 Ictero 1:400 Grippo 1:100 Bratis 1:6400 | | - |
| 3. Chance | Pom 1:3200 Ictero 1:200 Grippo 1:100 Bratis 1:1600 | Pom 1:3200 Ictero 1:200 Grippo 1:200 Bratis 1:1600 | Pom 1:3200 Ictero 1:400 Grippo 1:400. Harjo 1:100 Bratis 1:1600 | Pom 1:6400 Ictero 1:400 Grippo 1:400 Hardjo 1:100 Bratis 1:3200 | Pom 1:6400 Ictero 1:200 Grippo 1:400 Hardjo 1:100 Bratis 1:1600 | Pom 1:3200 Ictero 1:200 Grippo 1:200 Hardjo 1:200 Bratis 1:1600 | Pom 1:3200 Ictero 1:200 Grippo 1:200 Hardjo 1:200 Bratis 1:1600 | | Pom 1:3200; Ictero 1:400, Grippo 1:400, Harjo 1:200 Bratis 1:1600 |
| 4. Cobalt | Pom 1:800 Can 1:100 Ictero 1:400 Bratis 1:3200 | Pom 1:12,800 Can 1:100 Ictero 1:6400 Grippo 1:200 Hardjo 1:100 Bratis 1:12,800 | Pom 1:6400. Can 1:100 Ictero 1:6400 Grippo 1:100. Harjo 1:200 Bratis 1:12,800 | Pom 1:12,800 Ictero 1:3200 Grippo 1:100 Bratis 1:12,800 Hardjo 1:100 Can 1:100 | Pom 1:6400 Ictero 1:1600 Bratis 1:6400 | Pom 1:6400 Ictero 1:3200 Hardjo 1:100 Bratis 1:6400 | Pom 1:3200 Ictero 1:1600 Hardjo 1:100 Bratis 1:6400 | | Pom 1:6400; Can 1:400, Ictero 1:3200, grippo 1:100, Harjo 1:100, Bratis 1:6400 |
| 5. Jim Dandy (deceased) | Pom 1:3200 1:400 Ictero 1:800 bratislav | Pom 1:6400 Ictero 1:800 Grippo 1:100 Bratis 1:1600 | Pom 1:6400 Ictero 1:800 Grippo 1:100 Hardjo 1:100 Bratis 1:1600 | Pom 1:3200 Ictero 1:200 Grippo 1:100 Bratis 1:3200 | - | - | - | | - |
| 6. Judd | Pom 1:1600 1:400 Ictero 1:1600 Bratis | Pom 1:1600 Ictero 1:800 Bratis 1:1600 | Pom 1:1600 Ictero 1:400 Hardjo 1:100 Bratis 1:1600 | Pom 1:3200 Ictero 1:800 Hardjo 1:100 Bratis 1:1600 | Pom 1:800 Ictero 1:400 Bratis 1:800 | Pom 1:1600 Icteroe 1:400 Hardjo 1:100 Bratis 1:1600 | Pom 1:3200 Ictero 1:400 Bratis 1:1600 | | Pom 1:1600; Ictero 1:800, Bratis 1:3200 |
| 7. Lacy | Pom 1:3200 1:400 Ictero 1:1600 Bratis | Pom 1:800 Ictero 1:200 Bratis 1:800 | Pom 1:1600 Ictero 1:400 Hardjo 1:100 Bratis 1:1600 | Pom 1:3200 Ictero 1:400 Hardjo 1:100 Bratis 1:1600 | Pom 1:800 Ictero 1:200 Bratis 1:800 | Pom 1:3200 Icteroe 1:400 Hardjo 1:200 Bratis 1:3200 | Pom 1:3200 Ictero 1:400 Hardjo 1:100 Bratis 1:1600 | | Pom 1:1600; Ictero 1:400, Harjo 1:100, Bratis 1:3200 |
| 8. Red | - | Pom 1:3200 Ictero 1:400 Bratis 1:1600 | Pom 1:3200 Ictero 1:800 Hardjo 1:400 Bratis 1:3200 | Pom 1:3200 Ictero 1:400 Grippo 1:100 Hardjo 1:200 Bratis 1:3200 | Pom 1:3200 Ictero 1:400 Hardjo 1:200 Bratis 1:3200 | Pom 1:6400 Ictero 1:400 Hardj 1:400 Bratis 1:3200 | Pom 1:3200 Ictero 1:200 Hardjo 1:100 Bratis 1:1600 | | Pom 1:3200; Ictero 1:400; harjo 1:200, Bratis 1:1600 |
| 9. Rosen | Pom 1:800 1:200 Ictero 1:100 grippo. 1:1600 Bratis | Pom 1:800 Ictero 1:400 Grippo 1:200 Bratis 1:1600 | Pom 1:800 Ictero 1:200 Grippo 1:200 Hardjo 1:100 Bratis 1:1600 | Pom 1:800 Ictero 1:200 Grippo 1:200 Bratis 1:1600 | Pom 1:400 Ictero 1:200 Grippo 1:200 Bratis 1:1600 | Pom 1:800 Ictero 1:400 Grippo 1:200 Hardjo 1:200 Bratis 1:1600 | Pom 1:800 Ictero1:200 Grippo 1:200 Hardjo 1:100 Bratis 1:1600 | | Pom 1:400, Can 1:200, Ictero 1:400, Grippo 1:400, Bratis 1:1600 |
| 10. Slick | Pom 1:3200 Ictero 1:100 Grippo. 1:100 Bratis 1:1600 | Pom 1:3200 Ictero 1:400 Grippo 1:200 Bratis 1:3200 | Pom 1:1600 Ictero 1:200 Grippo 1:100 Hardjo 1:100 Bratis 1:3200 | - | Pom 1:1600 Ictero 1:200 Grippo 1:100 Bratis 1:1600 | Pom 1:3200 Ictero 1:200 Grippo 1:100 Hardjo 1:100 Bratis 1:1600 | Pom 1:3200 Ictero 1:200 Grippo 1:100 Hardjo 1:100 Bratis 1:1600 | | - |
| 11. Sundancer | Pom 1:3200 Ictero 1:800 Bratis 1:3200 | Pom 1:3200 Can 1:100 Ictero 1:800 Bratis 1:3200 | Pom 1:6400 Can 1:100 Ictero 1:1600 Bratis 1:6400 | Pom 1:6400 Can 1:100 Ictero 1:1600 Grippo 1:100 Bratis 1:6400 | Pom 1:3200 Ictero 1:800 Bratis 1:3200 | Pom 1:6400 Can 1:100 Icteroe 1:800 Bratis 1:6400 | Pom 1:3200 Can 1:100 Ictero 1:800 Bratis 1:3200 | | Pom 1:3200; Can 1:200; Ictero 1:1600; Bratis 1:6400 |
| 12.Trenche | Pom 1:1600 Ictero 1:400 Grippo. 1:200 Bratis 1:800 | Pom 1:3200 Can 1:200 Ictero 1:1600 Grippo 1:400 Bratis 1:3200 | Pom 1:3200 Can 1:100 Ictero 1:800 Grippo 1:400 Hardjo 1:200 Bratis 1:3200 | Pom 1:3200 Can 1:100 Ictero 1:800 Grippo 1:800 Hardjo 1:100 Bratis 1:3200 | Pom 1:1600 Ictero 1:800 Grippo 1:400 Hardjo 1:200 Bratis 1:3200 | Pom 1:3200 Icteroe 1:800 Grippo 1:400 Hardjo 1:100 Bratis 1:3200 | Pom 1:1600 Ictero 1:400 Grippo 1:400 hardjo 1:100 Bratis 1:1600 | | Pom 1:1600; Can 1:200; Ictero 1:800; Grippo 1:400; Harjo 1:100; Bratis 1:1600 |
| 13. Delila | - | - | Pom 1:1600 Ictero 1:400 Grippo 1:200 Bratis 1:1600 | Pom 1:3200 Ictero 1:400 Grippo 1:200 Bratis 1:1600 | Pom 1:1600 Ictero 1:200 Grippo 1:100 Bratis 1:800 | Pom 1:1600 Icteroe 1:400 Grippo 1:200 Hardjo 1:100 Bratis 1:1600 | - | |  |
| 14. Dupont (deceased) | Pom 1:1600 Ictero 1:200 Grippo 1:100 Bratis 1:1600 | Pom 1:3200 Ictero 1:200 Grippo 1:100 Bratis 1:1600 | Pom 1:1600. Ictero 1:200 Grippo 1:200. Harjo 1:100 Bratis 1:1600 | - | - | - | - | | - |
| 15. Hannah | Pom 1:200 Ictero 1:100 Grippo 1:400 Bratis 1:400 | Pom 1:200 Can 1:200 Ictero 1:100 Grippo 1:200 Bratis 1:400 | Pom 1:200 Can 1:200 Ictero 1:100 Grippo 1:200 Hardjo 1:100 Bratis 1:800 | Pom 1:400 Can 1:200 Ictero 1:100 Grippo 1:100 Hardjo 1:100 Bratis 1:800 | Pom 1:100 Can 1:100 Grippo 1:100 Bratis 1:400 | Pom 1:200 Can 1:200 Icteroe 1:100 Grippo 1:100 Hardjo 1:200 Bratis 1:800 | Pom 1:200 Can) 1:200 Grippo 1:100 Hardjo 1:100 Bratis 1:800 | | Pom 1:200; Can 1:400, Ictero 1:200, Grippo 1:200, Harjo 1:100, Bratis 1:800 |
| 16.Meara (deceased) | Pom 1:12800 Ictero 1:100 Bratis 1:800 | Pom 1:6400 Ictero 1:800 Bratis 1:3200 | Pom 1:12,800 Can 1:100 Ictero 1:800 Grippo 1:100 Hardjo 1:400 Bratis 1:6400 | - | - | - | - | | - |
| 17. Mo | Pom 1:400 Can 1:100 Bratis 1:200 | Pom 1:1600 Can 1:100 Ictero 1:200 Bratis 1:800 | Pom 1:800 Can 1:100 Ictero 1:200 Hardjo 1:100 Bratis 1:800 | Pom 1:800 Can 1:100 Ictero 1:100 Bratis 1:400 | Pom 1:400 Ictero 1:100 Bratis 1:400 | Pom 1:200 Ictero 1:200 Grippo 1:100 Bratis 1:1600 | Pom 1:800 Ictero 1:200 Hardjo 1:100 Bratis 1:800 | | Pom 1:800; Can 1:200; Ictero 1:200, Harjo 1:200, Bratis 1:1600 |

Pom: *L.* Pomona; Can: *L.* Canicola; Ictero: *L.* Icterohaemorrhagiae; Grippo: *L.* Grippotyphosa; Harjo: *L.* Harjo; Bratis: *L.* Bratislava

**S4 Table. Water sample leptospiral testing***

| **Source of water sample** | **Date of testing** | |
| --- | --- | --- |
|  | **December 2022** | **August 2024** |
| Well #1 | 86 | 2,871 |
| Well #2 | ND | ND |
| Creek | ND | 64,699 |
| Pond | 593 | 127 |
| County water | ND | 76 |

* cell equivalents/100 ml

ND = not detected

**S1 Fig. Water testing documentation (December 2022)S2 Fig. Water testing documentation (August 2024)**
